# Supplementary material for: Identification of Potential microRNA Panels for Male Non-Small Cell Lung Cancer Identification Using Microarray Datasets and Bioinformatics Methods
Source: J Pers Med. 2022 Dec 13;12(12):2056. doi: 10.3390/jpm12122056 (PMC9780989; doi:10.3390/jpm12122056)
Supplement: Supplementary file 1 [file jpm-12-02056-s001.zip › jpm-2017643-supplementary.pdf]

Supplementary Material

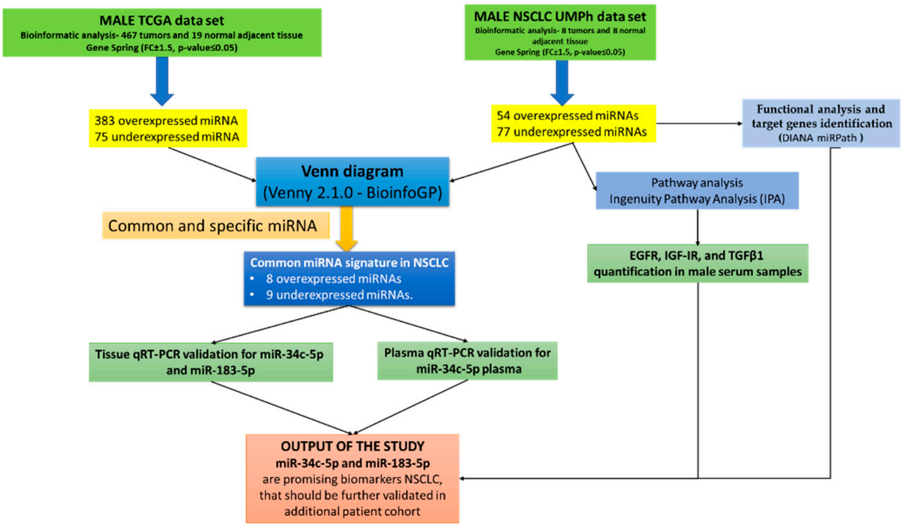

Figure S1. Workflow diagram presenting the study design**.** We used 2 datasets, TCGA and our cohort-generated microarray dataset, to identify specific sex-related dysregulated miRNAs. The clinical relevance of these miRNAs was evaluated using bioinformatics approaches and IPA analysis, which led to the selection of 3 target proteins and 2 miRNAs that were further validated on plasma and tissue samples. * UMPh—University of Medicine and Pharmacy "Iuliu-Hatieganu" Cluj-Napoca.

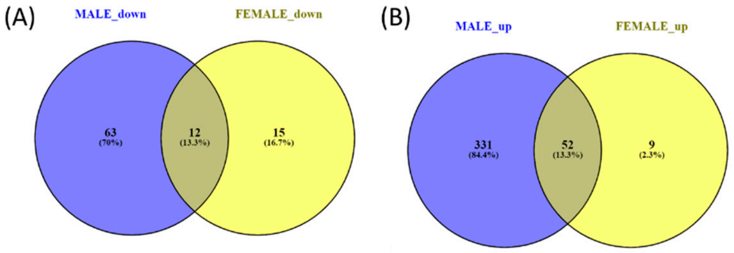

Figure S2. Venn diagram of the common and specific miRNA signature in male and female NSCLC-TCGA data set. (**A**) Underexpressed miRNAs in male and female samples. (**B**) Upregulated miRNAs in male and female models.

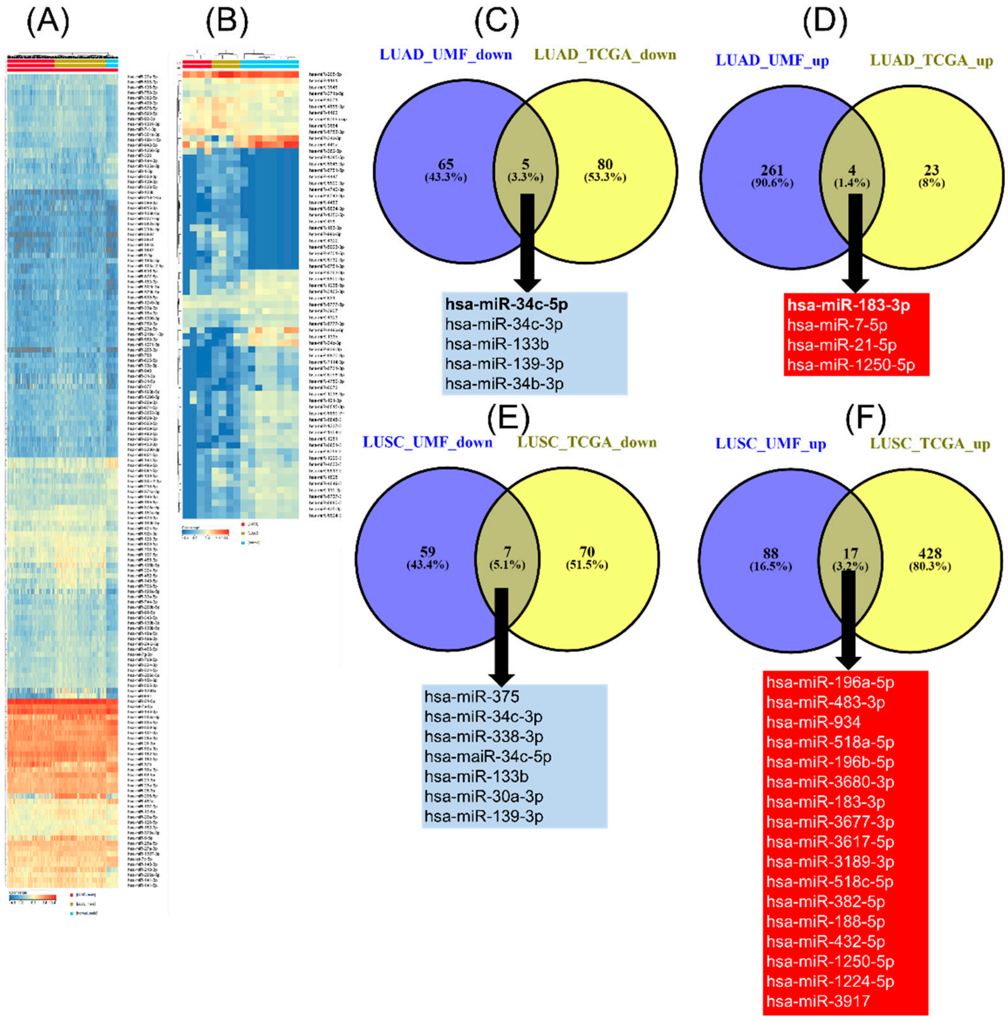

Figure S3. miRNA profiling in LUAD and LUSC male patients. (**A**) Heatmap for TGCA data, with Anova, *p*-value ≤ 0.05, emphasizes the differences among miRNA pattern in LUAD and LUSC versus normal adjacent tissue samples. (**B**) Heatmap for UMPh patient cohort, with Anova, *p*-value ≤ 0.05, emphasizes the differences among miRNA pattern in LUAD and LUSC versus normal adjacent tissue samples (the blue color is denoting underexpression, whereas the red color suggests overexpression). (**C**) Venn diagram for underexpressed miRNAs in LUAD for TCGA and UMPh cohort. (**D**) Venn diagram for overexpressed miRNAs in LUAD for TCGA and UMPh cohort. (**E**) Venn diagram for underexpressed miRNAs in LUSC for TCGA and UMPh cohort. (**F**) Venn diagram for overexpressed miRNAs in LUSC for TCGA and UMPh cohort.

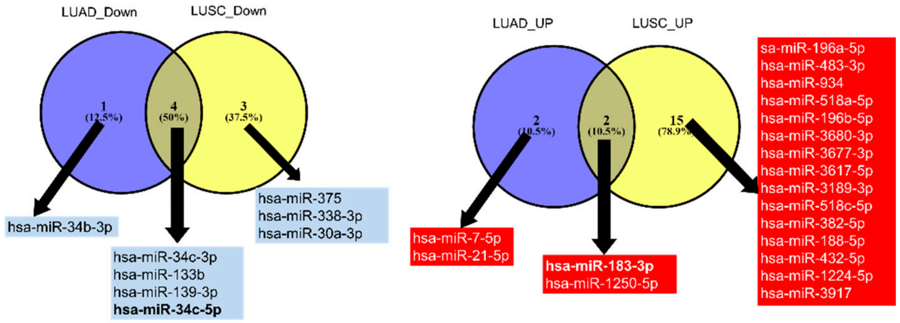

Figure S4. Venn diagram emphasizes common and specific miRNA signatures for LUAD and LUSC overlapping TCGA and our patient cohort dataset. (**A**) Downregulated miRNAs. (**B**) Upregulated miRNAs. miRNAs selected for validation are in bold.

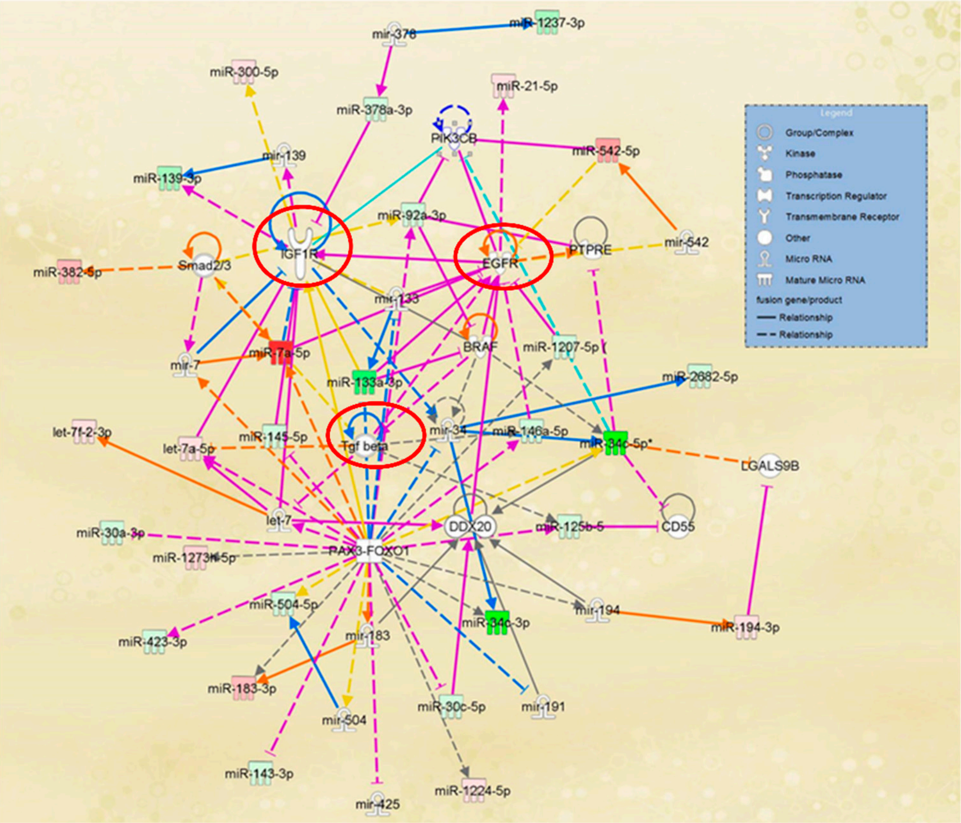

Figure S5. IPA network emphasizes the interconnection of miR-34 family members and miR-185-5p, with emphasis on critical elements EGFR, IGF1R, and TGFB1 (marked with a red circle); transcripts selected for additional validation at the protein level using ELISA.

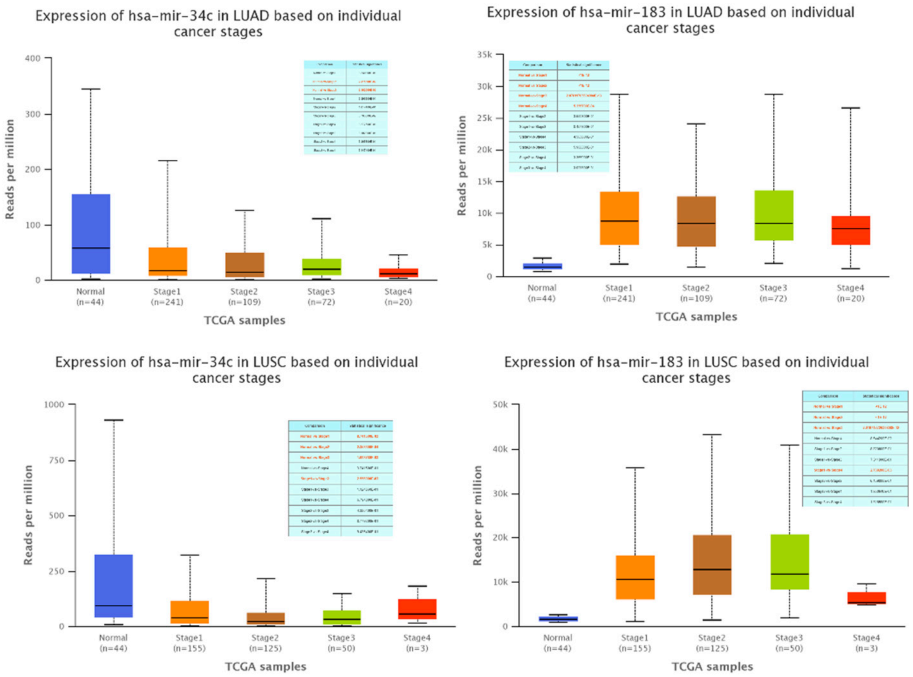

Figure S6. miR-34c and miR-183 expression levels on LUAD and LUSC on stages according to the UALCAN database.

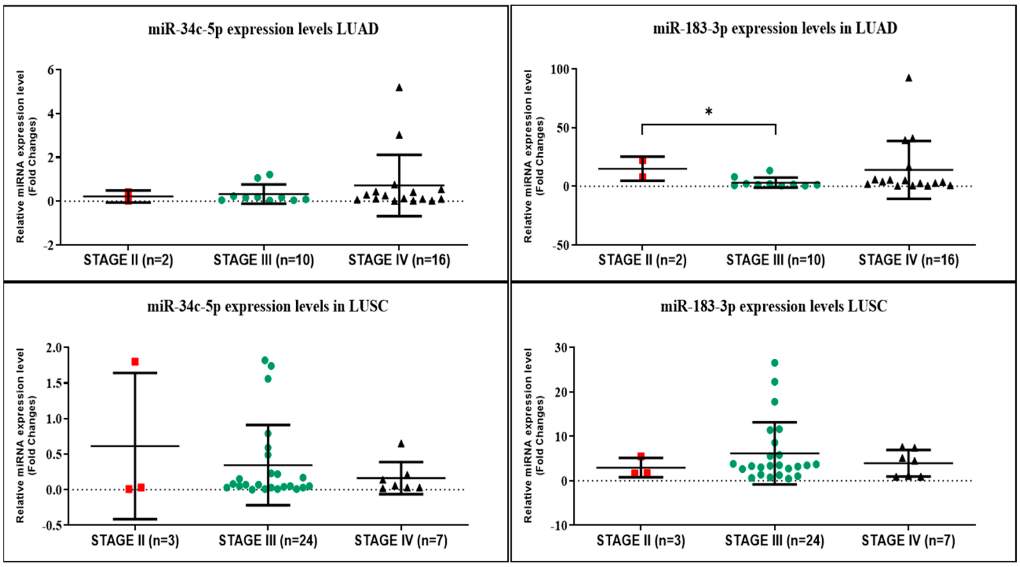

Figure S7. miR-34c and miR-183 expression levels on LUAD and LUSC on stages according to the qRT-PCR validation cohort.
